# Sensory satellite glial Gq-GPCR activation alleviates inflammatory pain via peripheral adenosine 1 receptor activation

**DOI:** 10.1038/s41598-020-71073-z

**Published:** 2020-08-25

**Authors:** Alison Xiaoqiao Xie, Aric Madayag, Suzanne K. Minton, Ken D. McCarthy, Anna P. Malykhina

**Affiliations:** 1 Department of Pharmacology, School of Medicine, University of North Carolina at Chapel Hill (UNC-CH), Chapel Hill, USA; 2 Division of Urology, Department of Surgery, University of Colorado Denver (UCD), Anschutz Medical Campus (AMC), 12700E 19th Ave., Room 6440D, Mail stop C317, Aurora, CO 80045 USA; 3 Department of Physiology and Biophysics, University of Colorado School of Medicine, 12700 East 19th Ave., Rm 6001, Mail Stop C317, Aurora, CO 80045 USA; 4 Professor Emeritus in the Department of Pharmacology, School of Medicine, University of North Carolina at Chapel Hill, 120 Mason Farm Road, 4010 Genetic Medicine Bldg, Campus Box 7365, Chapel Hill, NC 27599-7365 USA; 5 Present Address: Department of Surgery, UCD-AMC, Aurora, CO USA; 6 Present Address: NeuroCycle Therapeutics, Inc., 3829 N Cramer St., Shorewood, WI 53211 USA; 7 Present Address: Certara, 5511 Capital Center Drive, Ste. 204, Raleigh, NC 27606 USA

**Keywords:** Neuroscience, Cellular neuroscience

## Abstract

Glial fibrillary acidic protein expressing (GFAP^+^) glia modulate nociceptive neuronal activity in both the peripheral nervous system (PNS) and the central nervous system (CNS). Resident GFAP^+^ glia in dorsal root ganglia (DRG) known as satellite glial cells (SGCs) potentiate neuronal activity by releasing pro-inflammatory cytokines and neuroactive compounds. In this study, we tested the hypothesis that SGC Gq-coupled receptor (Gq-GPCR) signaling modulates pain sensitivity in vivo using *Gfap*-hM3Dq mice. Complete Freund’s adjuvant (CFA) was used to induce inflammatory pain, and mechanical sensitivity and thermal sensitivity were used to assess the neuromodulatory effect of glial Gq-GPCR activation in awake mice. Pharmacogenetic activation of Gq-GPCR signaling in sensory SGCs decreased heat-induced nociceptive responses and reversed inflammation-induced mechanical allodynia via peripheral adenosine A1 receptor activation. These data reveal a previously unexplored role of sensory SGCs in decreasing afferent excitability. The identified molecular mechanism underlying the analgesic role of SGCs offers new approaches for reversing peripheral nociceptive sensitization.

## Introduction

Chronic pain is closely associated with nociceptive sensitization and neuroinflammation^1,2^. In recent years, glial activation and glia-neuron interactions have emerged as key mechanisms underlying generation and maintenance of chronic pain^3^. Peripheral injury or inflammation induce long-lasting changes in GFAP^+^ glia including structural reorganization^4,5^, cell proliferation^6,7^, changes to neurotransmitter scavenging^8–11^, ion buffering capacities^12^, increases in gap junction coupling^13–15^, and release of pro-inflammatory cytokines, neurotrophins, and neuroactive compounds^3,16–19^. These changes in GFAP^+^ glia occur throughout the entire nociceptive pathway, including peripheral nerves^20,21^ and higher integrative brain regions^22^. Pharmacological inhibition of GFAP^+^ glial activation reduced manifestation of pain in animal models^23,24^. Therefore, activation of GFAP^+^ glia during inflammatory pain is considered to be pro-nociceptive and exacerbates symptoms of chronic pain.

Despite the growing body of work on glia-neuron interactions in nociceptive pathways, the hypothesis that GFAP^+^ glial Gq-GPCR activation modulates nociception in vivo has not been directly tested. This is largely due to limited means to selectively manipulate GFAP^+^ glial signaling in vivo. To test this hypothesis, we employed a transgenic mouse expressing the Gq-coupled DREADD (hM3Dq) under control of a GFAP minimal promoter (*Gfap*-hM3Dq mice), as previously described^25^. The hM3Dq-HA immunoreactivity was restricted to GFAP^+^ glia in both CNS and PNS^25^. Clozapine-*N*-oxide (CNO), the agonist for hM3Dq receptors, induces intracellular Ca^2+^ elevations comparable to those evoked by endogenous Gq-GPCR agonists in a majority of GFAP^+^ glia in the brain (CA1 hippocampal astrocytes^25^, visual cortical astrocytes^26,27^, medulla astrocytes^27^, and in PNS superior cervical ganglia satellite glial cells^27^ and enteric glia^28^). These data demonstrated the functional expression and integration of hM3Dq in GFAP^+^ glial signaling pathways in *Gfap*-hM3Dq mice. In addition, the hM3Dq exhibits no constitutive activity and can only be activated by the bio-inert small molecule, CNO^29^, providing a unique model for assessing the role of GFAP^+^ glia Gq-GPCR signaling in pain modulation in free-moving animals.

The present study sought to identify glial Gq-GPCR signaling as a viable therapeutic target for ameliorating chronic and acute pain. Using a CFA-induced inflammatory pain model, we demonstrated that the activation of Gq-GPCR signaling pathways in sensory ganglionic SGCs induced robust and long-lasting (~ 90 min) analgesia in the hind paws of *Gfap*-hM3Dq mice. The analgesic effects were observed not only in CFA-injected hind paws which suffered from inflammation-induced hypersensitivity, but also in saline-injected control (Von Frey tests) and intact (Hargreaves tests) hind paws. Pharmacological experiments and the usage of adenosine receptor knockout mice revealed that the analgesic effect of glial hM3Dq activation was due to the activation of Gq-GPCR signaling pathways in sensory SGCs, and was dependent on adenosine A1 receptor (A_1_R) activation in the PNS. These data revealed a novel role of sensory SGC Gq-GPCR signaling in pain control, suggesting a strong therapeutic potential of targeting the peripheral glial signaling for the treatment of acute and chronic pain. Future studies are warranted to develop selective manipulation of sensory SGC signaling as an alternative approach in pain management.

## Results

### Pharmacogenetic activation of Gq-GPCR signaling pathways in GFAP^+^ glia alleviated inflammation-induced mechanical allodynia in mice

**Figure 1:**
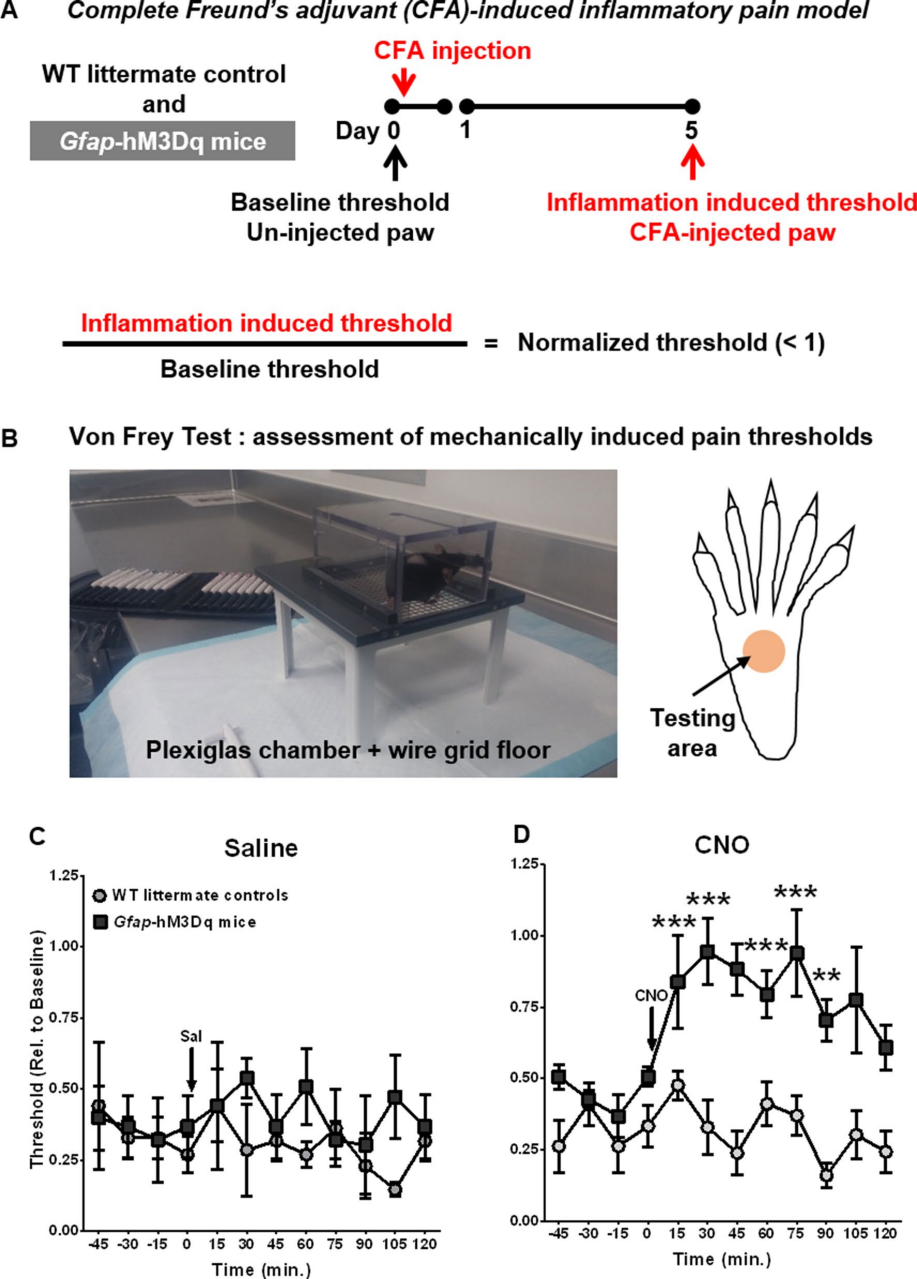
CNO-induced GFAP^+^ glial Gq-GPCR activation alleviated CFA-induced mechanical allodynia in vivo. (**A**) Schematic timeline of CFA-induced inflammatory pain animal model; (**B**) Von Frey apparatus for assessing mechanically induced pain thresholds. (**C**) CFA injection into hind paw induced comparable levels of mechanical allodynia in both *Gfap*-hM3Dq mice and WT littermate controls (N = 3 per genotype). (**D**) 0.25 mg/kg CNO i. p. administration largely reversed CFA-induced mechanical allodynia in CFA-injected paws in *Gfap*-hM3Dq mice, but not in WT littermate controls (N = 5 per genotype); **p < 0.01; ***p < 0.001 compared to pre-CNO. (**B**) was taken by Alison Xie; (**C**) and (**D**) were prepared using Graphpad Prism 7.04.

**Figure 2:**
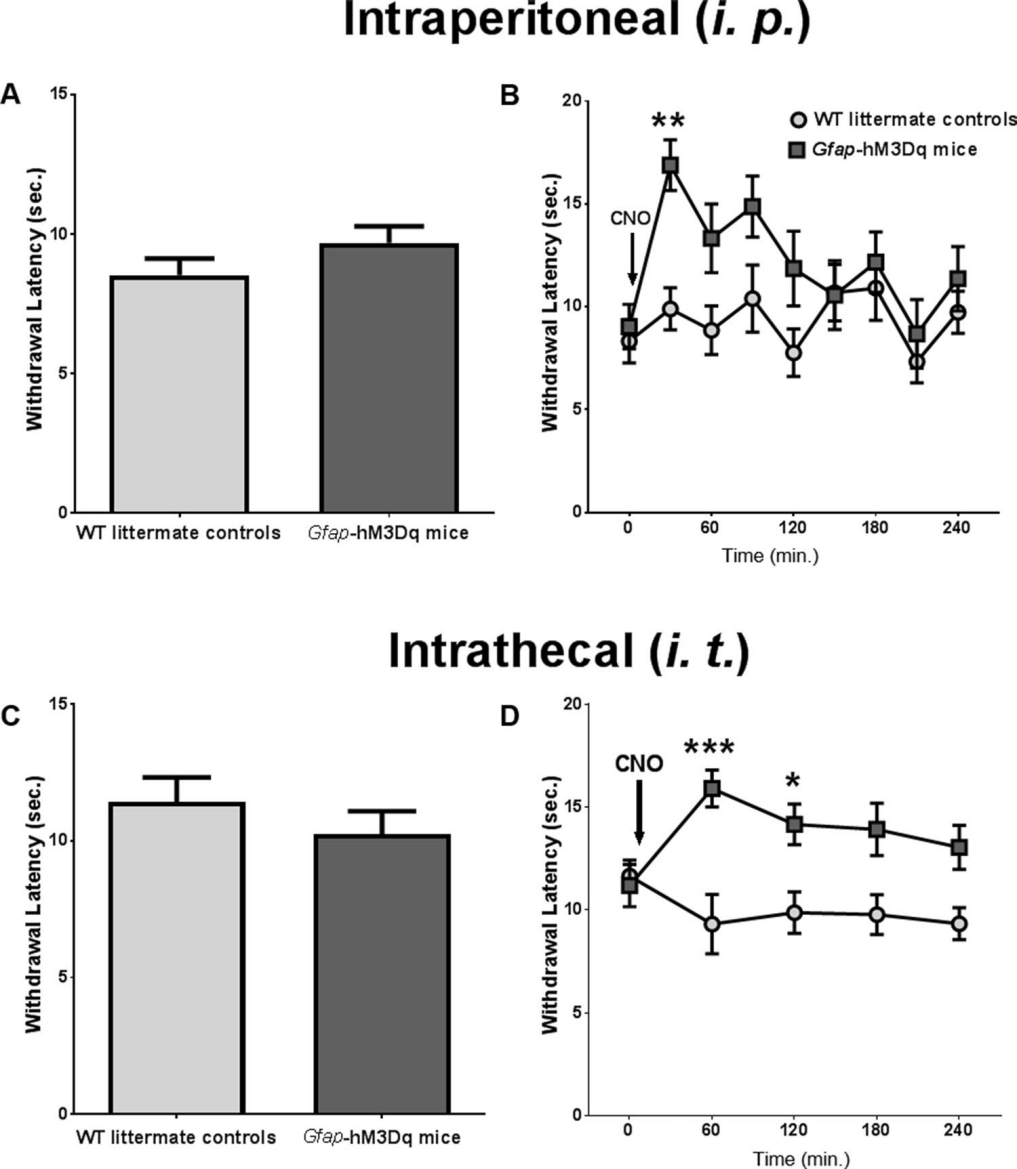
CNO-induced GFAP^+^ glial Gq-GPCR activation decreased heat sensitivity in the hind paws of naive mice. (**A**) Baseline heat sensitivity without any drug administration of animals used in (**B**). *Gfap*-hM3Dq mice exhibited similar heat sensitivity compared to their WT littermate controls in Hargreaves test (N = 9 control, N = 10 *Gfap*-hM3Dq). (**B**) 0.25 mg/kg CNO i. p*.* administration significantly increased paw withdrawal frequency at 30 min after CNO delivery (N = 9 control, N = 10 *Gfap*-hM3Dq). (**C**) Baseline heat sensitivity without any drug administration of animals used in (**D**). *Gfap*-hM3Dq mice exhibited similar heat sensitivity compared to their WT littermate controls (N = 10 control, N = 17 *Gfap*-hM3Dq). (**D**) Intrathecal administration (i. t.) of 0.5 mg/kg CNO significantly increased paw withdrawal latency up to 2 h (N = 10 control, N = 17 *Gfap*-hM3Dq). In both (**B**) and (**D**), *p < 0.05; **p < 0.01; ***p < 0.001 compared to pre-CNO. Figures were prepared using Graphpad Prism 7.04.

To test our hypothesis that Gq-GPCR signaling in GFAP^+^ glia modulates pain sensitivity, *Gfap*-hM3Dq mice^25^ were used to selectively activate GFAP^+^ glial Gq-GPCR signaling in vivo*.* We first tested our hypothesis in the CFA-induced inflammatory pain model. *Gfap*-hM3Dq mice and their WT littermate controls were subjected to unilateral injection of CFA in right hind paws (Fig. 1A). Mechanical pain sensitivity in CFA-injected hind paws was assessed prior to (baseline threshold) and five days after CFA injection (inflammation-induced threshold); normalized threshold was then calculated (Fig. 1B). CFA induced well-documented mechanical hyperalgesia and mechanical allodynia^30^ in CFA-injected paw in both the *Gfap*-hM3Dq mice and their littermate controls, as evidenced by a decreased mechanical pain threshold (~ 40% when compared to the baseline threshold measured from the same paw) (Fig. 1C). There was no significant difference in the reduction of mechanical pain thresholds between *Gfap*-hM3Dq mice and their littermate controls (N = 3 per group). A single administration of CNO (0.25 mg/kg, i. p.) reversed the normalized mechanical pain threshold in *Gfap*-hM3Dq mice but not in littermate controls (Fig. 2D). The CNO-induced reversal of inflammatory allodynia occurred within 15 min after drug administration and persisted for nearly 90 min. CNO injection resulted in significant main effects of time (F_11,88_ = 3.86, p < 0.001), genotype (F_1,8_ = 36.13, p < 0.001), as well as a significant time-by-genotype interaction (F_11,88_ = 3.22, p < 0.01). Conversely, saline exposure resulted in no significant main effects of time (F_11,44_ = 0.39), genotype (F_1,4_ = 1.36), and no significant time-by-genotype interaction (F_11,44_ = 0.54). These data suggested that Gq-GPCR activation in GFAP^+^ glia substantially alleviated inflammation-induced mechanical allodynia.

### Gq-DREADD mediated Gq-GPCR activation in GFAP^+^ glia decreased hind paw heat sensitivity in naive mice

We next tested if CNO-induced reduction of pain sensitivity was restricted to mechanical nociception. We chose to examine effects of glial Gq-GPCR activation on heat-induced pain sensitivity, which is mechanistically distinct from mechanical nociception and predominately mediated by different nociceptive nerve fibers^31^. Hargreaves test was used to assess heat sensitivity in the hind paws of *Gfap*-hM3Dq mice and WT littermate controls. In all experiments, a fixed low intensity thermal beam was applied to a hind paw and the paw withdrawal latency to the beam was recorded. There was no significant difference in paw withdrawal latency between *Gfap*-hM3Dq mice and littermate controls (Fig. 2A). However, CNO (0.5 mg/kg, i. p.) significantly increased paw withdrawal latency of *Gfap*-hM3Dq mice, but not of littermate controls (Fig. 2B). Significant main effects of time (F_8,136_ = 3.73, p < 0.001) and genotype (F_1,17_ = 6.58, p < 0.05) were observed, but no significant time-by-genotype interaction (F_8,136_ = 1.70). These data demonstrated that Gq-GPCR activation in GFAP^+^ glia reduced hind paw heat sensitivity in addition to mechanical sensitivity, suggesting a general analgesic effect of glial Gq-GPCR activation.

### Gq-GPCR activation in peripheral SGCs was responsible for CNO-induced decreases in afferent sensitivity

Systemic administration of CNO activates GFAP^+^ glial Gq-GPCR signaling in both CNS astrocytes and PNS glia^27^. To identify the site of the analgesic effect of glial Gq-GPCR activation, CNO (0.5 mg/kg) was delivered via i.t. injections in *Gfap*-hM3Dq mice and their WT littermate controls (control: N = 10, *Gfap*-hM3Dq: N = 17). A single i.t. injection of CNO mimicked the analgesic effect on hind paw heat sensitivity in *Gfap*-hM3Dq mice, suggesting that Gq-GPCR activation in spinal cord astrocytes and/or sensory satellite glial cells^32^ is sufficient to modulate pain sensitivity in vivo (Fig. 2D). There was no significant main effect of time (F_4,100_ = 0.87) but a significant main effect of genotype (F_1,25_ = 9.40, p < 0.01), and a significant time-by-genotype interaction (F_4,100_ = 4.66, p < 0.01) were observed. There was no significant difference in baseline paw withdrawal latency between *Gfap*-hM3Dq mice and the littermate controls (Fig. 2C).

**Figure 3:**
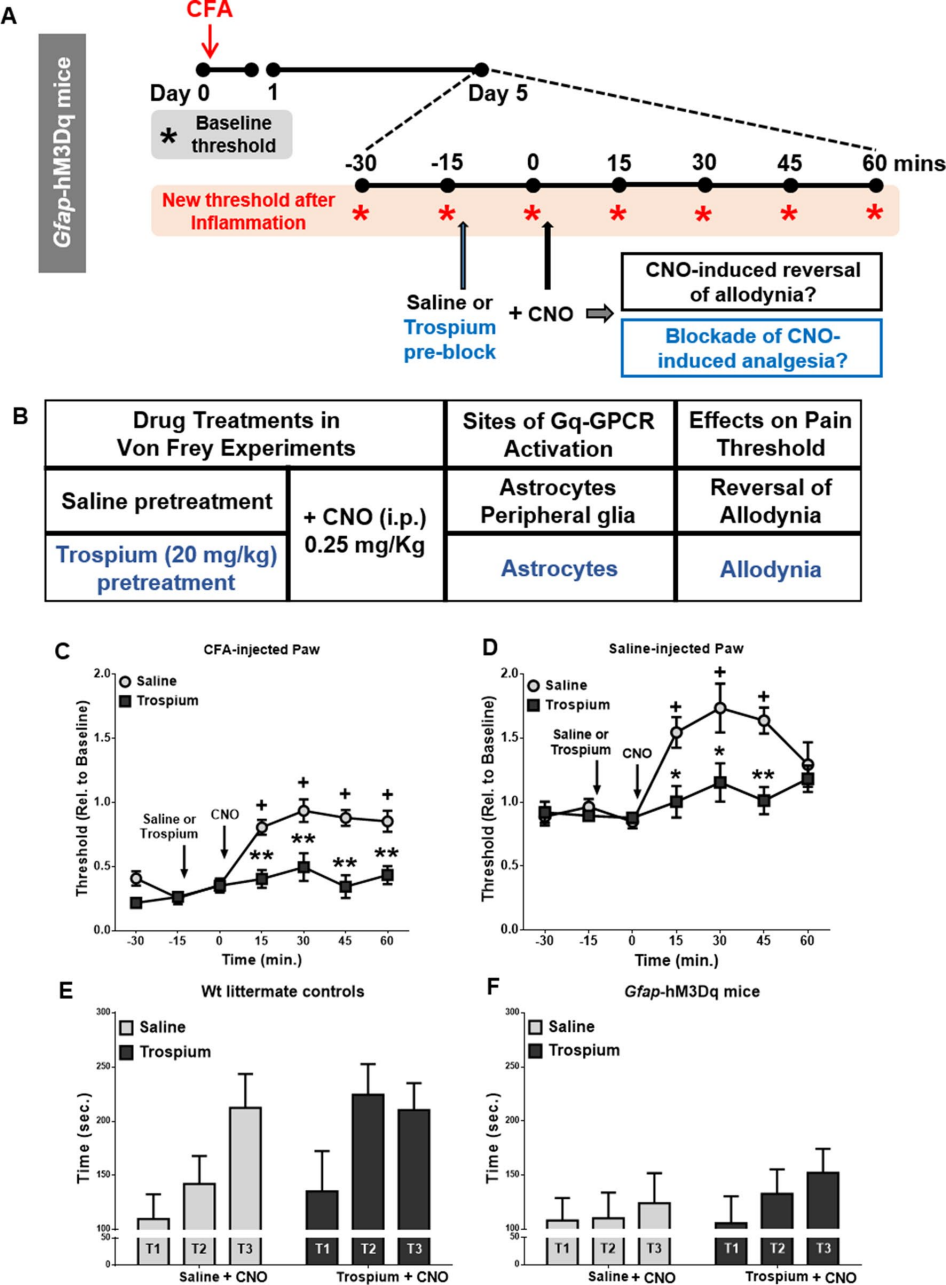
CNO-induced analgesia in *Gfap*-hM3Dq mice was due to Gq-GPCR activation in peripheral GFAP^+^ glia and not due to CNO-induced decreases in motor activity. (**A**) Schematic timeline of peripheral hM3Dq blockade experiments *Gfap*-hM3Dq mice were subjected to CFA-induced inflammatory pain model as in Fig. 1. Von Frey tests were used to assess both baseline and inflammation induced mechanical pain threshold in both CFA-injected and saline-injected hind paws (N = 21 mice). (**B**) A summary of peripheral Gq-GPCR activation blockade in *Gfap*-hM3Dq mice with saline or Trospium pretreatment. (**C**,**D**) Trospium pretreatment prevented CNO-induced increases of pain threshold in both CFA-injected (**C**) and saline-injected (**D**) hind paws in *Gfap*-hM3Dq mice (N = 11 saline pretreated, N = 10 trospium pretreated). (**E**) 0.25 mg/kg CNO i. p. administration had no effect on rotarod performance (three trials, T1, T2, and T3) in WT littermate control mice, nor did pretreatment with trospium (N = 6 per group). (**F**) CNO led to decreased rotarod performance in *Gfap*-hM3Dq mice. Trospium pretreatment did not alter CNO-induced decreases in rotarod performance (N = 5 per group). ^+^p < 0.01 compared to baseline in saline-treated mice; *p < 0.01, **p < 0.001 compared to saline-treated mice. (**C**–**F**) Were prepared using Graphpad Prism 7.04.

To distinguish the possible analgesic effects of Gq-GPCR activation between spinal astrocytes and sensory satellite glial cells, trospium chloride, a peripheral muscarinic receptor blocker, was used to inhibit CNO-induced hM3Dq activation in PNS glia^27^. Using the CFA-induced inflammatory pain model (Fig. 3A), *Gfap*-hM3Dq mice were pretreated with either trospium chloride (20 mg/kg, i. p.) or saline 15 min prior to CNO administration (0.5 mg/kg, i. p.) followed by Von Frey testing (Fig. 3B; N = 11 saline pretreated; N = 10 trospium pretreated). Normalized paw withdrawal thresholds of both CFA- and saline-injected paws were calculated. Saline treatment did not alter CNO-induced reduction of mechanical allodynia in CFA-injected hind paws in *Gfap*-hM3Dq mice (Fig. 3C). In contrast, pretreatment with trospium chloride completely blocked CNO-induced analgesic effects in CFA-injected paws (Fig. 3C), suggesting that peripheral activation of hM3Dq was necessary for the CNO-induced analgesic effect in *Gfap*-hM3Dq mice. In the CFA-injected hind paws, we observed main effects of trospium (F_1,19_ = 25.31, p < 0.0001), time (F_6,114_ = 20.17, p < 0.0001), and a significant trospium-by-time interaction (F_6,114_ = 7.19, p < 0.0001).

Interestingly, CNO also induced an analgesic effect in the saline-injected paws (Fig. 3D). In absence of inflammation, the mechanical pain thresholds in the saline-injected paws (left hind paws) remained unchanged at five days post-CFA injection into the right hind paws. CNO administration (0.25 mg/kg, i. p.) increased the mechanical pain threshold in saline-injected paws by approximately 50% during the first hour after CNO administration, similar to the observed effects on the CFA-injected paws. These data suggest that glial activation-induced analgesia was independent of CFA-induced inflammation. Pretreatment with trospium chloride also blocked CNO-induced analgesic effect in saline-injected paws in *Gfap*-hM3Dq mice (Fig. 3D; N = 11 saline pretreated, N = 10 trospium pretreated), further confirming that peripheral GFAP^+^ glial Gq-GPCR activation was responsible for CNO-induced decreases in pain sensitivity in *Gfap*-hM3Dq mice. For the saline-injected paw, a two-way ANOVA revealed significant main effects of trospium chloride (F_1,19_ = 9.05, p < 0.01 to saline pre-treatment), time (F_6,114_ = 11.03, p < 0.0001), and a significant trospium-by-time interaction (F_6,114_ = 4.52, p < 0.001).

CNO-induced activation of GFAP^+^ glial Gq-GPCR signaling produces mild sedative effects and decreases motor performance^25^. To determine if trospium blockade of CNO-induced analgesia was due to a reversal of locomotor deficits following CNO administration, *Gfap*-hM3Dq mice and their wild-type littermate controls were subjected to rotarod tests during which trospium and CNO were administered. Both *Gfap*-hM3Dq mice and their littermate controls received either saline or trospium (20 mg/kg, i. p.) pretreatment 15 min prior to CNO administration (0.25 mg/kg, i. p.). CNO administration slightly decreased rotarod performance in *Gfap*-hM3Dq mice (Fig. 3F) but not in littermate controls (Fig. 3E), consistent with our previous report^25^. Trospium had no additional effect on CNO-induced decreases in rotarod performance in *Gfap*-hM3Dq mice (Fig. 3F; N = 5 per group), suggesting that CNO-induced analgesia was not secondary to CNO-induced motoric deficits. There were no significant main effects of trospium (F_1,8_ = 1.00), trial (F_2,16_ = 0.75), nor a significant trospium-by-trial interaction (F_2,16_ = 0.20). Neither CNO nor trospium pretreatment produced any effect on rotarod performance in WT mice (Fig. 3E, N = 6 per group). There was no main effect of trospium (F_1,10_ = 1.40), a main effect of trial (F_2,20_ = 7.19, p < 0.01), and no significant trospium-by-trial interaction (F_2,20_ = 1.62).

Together, these data strongly suggest that Gq-GPCR activation in sensory ganglionic SGCs is responsible for CNO-induced decreases in pain sensitivity.

### Sensory SGC Gq-GPCR activation underlies CNO-induced analgesia

**Figure 4:**
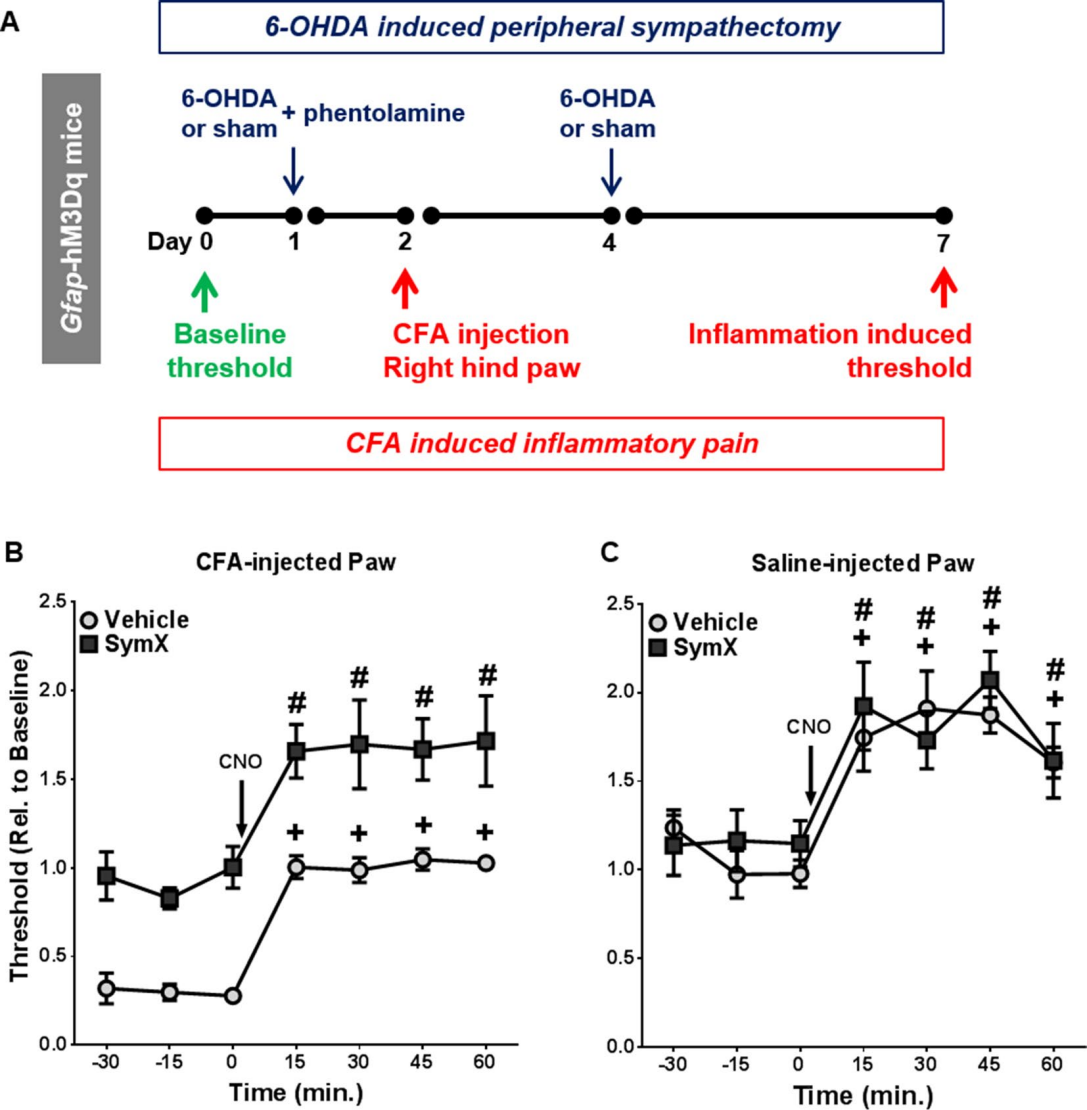
Peripheral chemical sympathectomy reversed inflammation induced mechanical allodynia in CFA-injected hind paws in *Gfap*-hM3Dq mice, while CNO induced additional analgesia effects in both CFA-injected and saline-injected hind paws. (**A**) Experimental timeline of 6-OHDA induced peripheral chemical sympathectomy and CFA-induced inflammatory pain in *Gfap*-hM3Dq mice (N = 6 vehicle injection, N = 5 6-OHDA injection). (**B**) Peripheral sympathectomy blocked CFA-induced mechanical allodynia in *Gfap*-hM3Dq mice. CNO-induced analgesia was observed in CFA-injected hind paws in both the vehicle- and 6-OHDA-injected groups. (**C**) Sympathectomy does not affect either the baseline pain threshold or CNO-induced analgesia in saline-injected hind paw. ^+^p < 0.01 compared to baseline in vehicle treated mice; ^#^p < 0.01 compared to baseline in sympathectomized mice. (**B**,**C**) Were prepared using Graphpad Prism 7.04.

Administration of CNO in *Gfap*-hM3Dq mice also induces Gq-GPCR activation in sympathetic ganglia, which increases sympathetic drive to the peripheral organs^27^. Sympathetic activation has been shown to exacerbate pain sensation via activation of adrenergic receptors on sensory nerves^33–35^. To test the extent to which CNO-induced sympathetic activation contributes to CNO-induced changes in pain sensitivity, *Gfap*-hM3Dq mice were subjected to 6-OHDA induced peripheral chemical sympathectomy (SymX)^27,36^ (Fig. 4A). When injected in the periphery, 6-OHDA enters noradrenergic terminals via norepinephrine reuptake transporters and destroys sympathetic terminals^37^, sparing cholinergic neurons, Schwann cells, non-myelinating glia including ganglionic SGCs, endothelial cells, and adrenal medulla^37,38^. In the CFA-injected hind paws, 6-OHDA-induced sympathectomy completely prevented the development of CFA-induced mechanical allodynia (Fig. 4B,C, N = 5 6-OHDA injection, N = 6 vehicle injection), but did not alter the baseline mechanical sensitivity in the saline-injected paws. There was a main effect of SymX (F_1,9_ = 24.85, p < 0.001), time (F_6,54_ = 43.18, p < 0.0001), but no SymX-by-time interaction (F_6,54_ = 0.3026). These data implied that sympathetic mechanisms are responsible for CFA-induced hypersensitivity in the used animal model. Peripheral sympathectomy did not result in changes in baseline pain threshold in saline-injected hind paws (Fig. 4C). There was no main effect of SymX (F_1,9_ = 0.18), a main effect of time (F_6,54_ = 20.78, p < 0.0001), and no significant SymX-by-time interaction (F_6,54_ = 0.8228). Application of CNO (i. p.) led to additional analgesic effects on both the CFA- and saline-injected paws in *Gfap*-hM3Dq mice. However, the CNO-induced analgesic effect was not altered by peripheral sympathectomy (Fig. 4B,C, N = 5 6-OHDA injection, N = 6 vehicle injection). These data provided evidence that while CFA-induced hind paw mechanical allodynia was dependent on sympathetic mechanisms, glial Gq-GPCR activation-evoked analgesia was not. Moreover, these data strongly suggested that sensory SGCs, but not sympathetic SGCs, were responsible for CNO-induced analgesia in *Gfap*-hM3Dq mice.

### Sensory SGC Gq-GPCR activation led to mechanical analgesia via peripheral A_1_R activation

**Figure 5:**
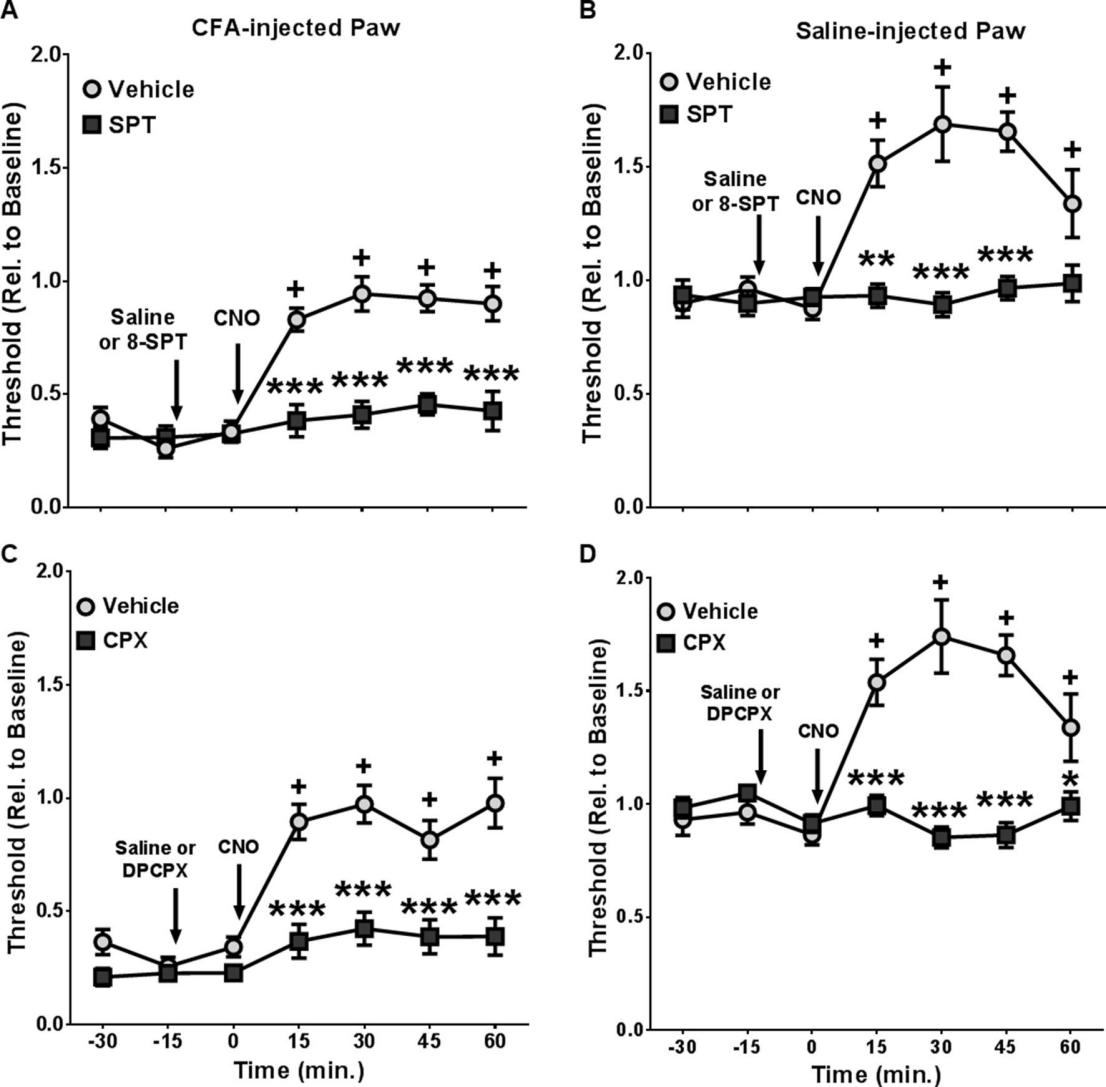
Pharmacological blockade of peripheral A_1_ Adenosine receptors prevented CNO-induced analgesia in *Gfap*-hM3Dq mice. Peripheral administration of SPT, a blood brain barrier impermeable inhibitor of adenosine receptors ablated CNO-induced analgesia in CFA-injected (**A**) and saline-injected (**B**) hind paws (N = 13 saline treated, N = 5 SPT treated). A_1_ adenosine receptor blocker CPX also prevented CNO-induced analgesia in CFA-injected (**C**) and saline-injected (**D**) hind paws (N = 13 saline treated, N = 12 CPX treated). ^+^p < 0.01 compared to baseline in saline-treated animals; *p < 0.05, **p < 0.01, ***p < 0.001 compared to saline-treated animals. Figures were prepared using Graphpad Prism 7.04.

**Figure 6:**
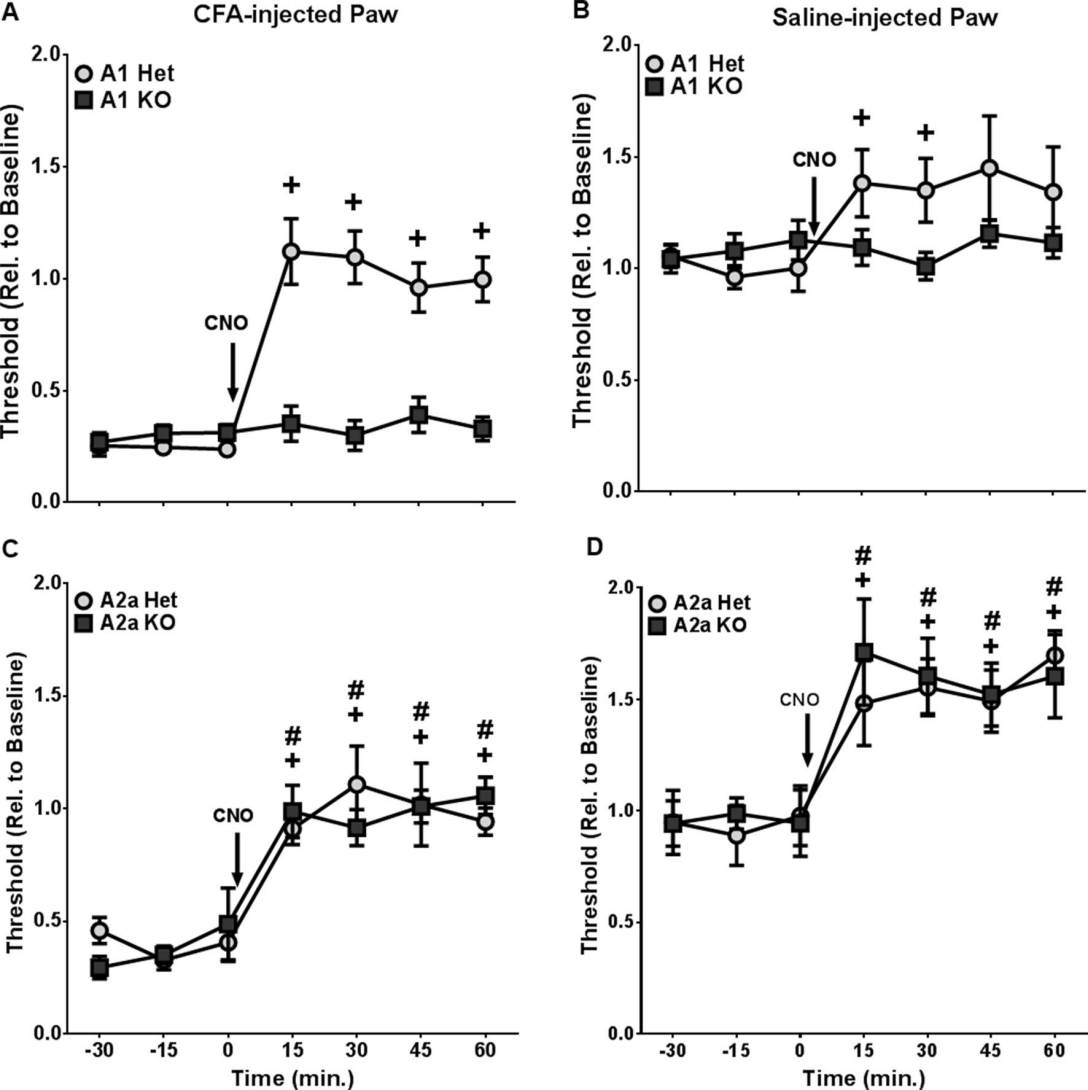
*Gfap*-hM3Dq mice lacking A_1_R, but not those lacking A_2A_R failed to exhibit CNO-induced analgesia. CNO-induced analgesia was observed in A_1_ heterozygous (Het) but not in A_1_ knockout (KO) mice in both CFA-injected (**A**) and saline-injected (**B**) hind paws (N = 9 A_1_ Het mice, and N = 13 A_1_ KO mice). CNO-induced analgesia remained intact in both A_2A_ heterozygous and in A_2A_ knockout mice in both the CFA-injected (**C**) and saline-injected (**D**) hind paws (N = 7 per group). ^+^p < 0.01 compared to baseline in heterozygous mice; ^#^p < 0.01 compared to baseline in knockout mice. Figures were prepared using Graphpad Prism 7.04.

Thus far, our data suggested that Gq-GPCR activation in SGCs in the peripheral sensory ganglia produced strong analgesia in both intact hind paws (Figs. 2, 3D, 4C, 5B,D, 6B,D) and hind paws with inflammation-induced hypersensitivity (Figs. 1, 3C, 4B, 5A,C, 6A,C). Previous reports indicated that sensory SGCs modulate peripheral neuronal activity via purinergic mechanisms in the context of neuropathic^39^ and inflammatory^17^ pain. To test if CNO-induced analgesia in *Gfap*-hM3Dq mice was dependent on glia-neuron adenosine signaling in sensory ganglia, pharmacological and genetic approaches were employed to dissect the potential molecular mechanism.

*Gfap*-hM3Dq mice were subjected to CFA-induced inflammatory pain, as described in Fig. 1A. Von Frey tests were performed prior to and 5 days after CFA-injections on both CFA-injected and saline-injected hind paws to assess the mechanical pain threshold. Two adenosine receptor blockers were used in separate experiments to evaluate the potential role of adenosine receptors in CNO-induced analgesia. In one set of experiments, 8-(*p*-Sulfophenyl) theophylline hydrate (SPT), a non-selective adenosine receptor antagonist that does not readily cross the blood–brain barrier^40^ was injected (10 mg/kg, i.p.) in *Gfap*-hM3Dq mice 15 min prior to CNO administration. In both CFA-injected and saline-injected hind paws, pretreatment with SPT completely blocked CNO-induced analgesia in *Gfap*-hM3Dq mice (Fig. 5A,B, N = 13 saline treated, N = 5 SPT treated). For the CFA-treated paw, a two-way ANOVA with time as a repeated measure and SPT as a between subjects factor revealed main effects of SPT (F_1,16_ = 23.59, p < 0.001), time (F_6,96_ = 16.32, p < 0.0001), and a significant SPT-by-time interaction (F_6,96_ = 7.78, p < 0.0001). For the saline-injected paw, main effects were found for SPT (F_1,16_ = 12.81, p < 0.01), time (F_6,96_ = 5.42, p < 0.0001), and a significant SPT-by-time interaction (F_6,96_ = 5.11, p < 0.001). These data suggested that activation of peripheral adenosine receptors was necessary for CNO-induced analgesia in *Gfap*-hM3Dq mice.

In the second set of experiments, 8-Cyclopentyl-1,3-dipropylxanthin (CPX), a selective A_1_R antagonist was administered (1 mg/kg, i. p.) 15 min prior to CNO administration (Fig. 5C,D). Pretreatment with CPX also blocked CNO-induced analgesia in both CFA-injected and saline-injected hind paws of *Gfap*-hM3Dq mice (Fig. 5C,D, N = 13 saline treated, N = 12 CPX treated). In the CFA-treated paw, we found main effects of CPX (F_1,23_ = 45.34, p < 0.0001), time (F_6,138_ = 21.47, p < 0.0001), and a significant CPX-by-time interaction (F_6,138_ = 6.87, p < 0.0001). For the saline-injected paw, we observed significant main effects of CPX (F_1,23_ = 28.92, p < 0.0001), time (F_6,138_ = 9.71, p < 0.0001), and a significant CPX-by-time interaction (F_6,138_ = 15.07, p < 0.0001). Taken together, these data suggested that activation of peripheral A_1_Rs was necessary for peripheral glial activation-induced analgesia.

We next tested the role of A_1_R activation in CNO-induced analgesia by genetically deleting A_1_Rs or A_2A_Rs in *Gfap*-hM3Dq mice. Genetic deletion of A_1_R prevented CNO-induced analgesia (Fig. 6A,B). For the CFA treated paw, a two-way genotype-by-time ANOVA revealed main effects of genotype (F_1,120_ = 32.88, p < 0.0001), time (F_6,120_ = 26.44, p < 0.0001) and a genotype-by-time interaction (F_6,120_ = 21.48, p < 0.0001). For the saline-injected paw, we observed no main effect of genotype (F_1,120_ = 1.48), a main effect of time (F_6,120_ = 3.82, p < 0.01), and a significant genotype-by-time interaction (F_6,120_ = 3.23, p < 0.01).

In contrast, genetic deletion of A_2A_ receptors had no effect on CNO-induced analgesia in *Gfap*-hM3Dq mice (Fig. 6C,D). For the CFA-injected paw, there was only a main effect of time (F_6,72_ = 30.50, p < 0.0001), but no main effect of genotype (F_1,72_ = 0.01), and no significant genotype-by-time interaction (F_6,72_ = 1.07). Further, for the saline-injected paw, only a main effect of time was observed (F_6,72_ = 17.61, p < 0.0001), with no main effect of genotype (F_1,72_ = 0.07) nor a significant genotype-by-time interaction (F_6,72_ = 0.40). Collectively, these data suggest that sensory SGC Gq-GPCR-mediated analgesia was dependent on peripheral A_1_R activation.

## Discussion

**Figure 7:**
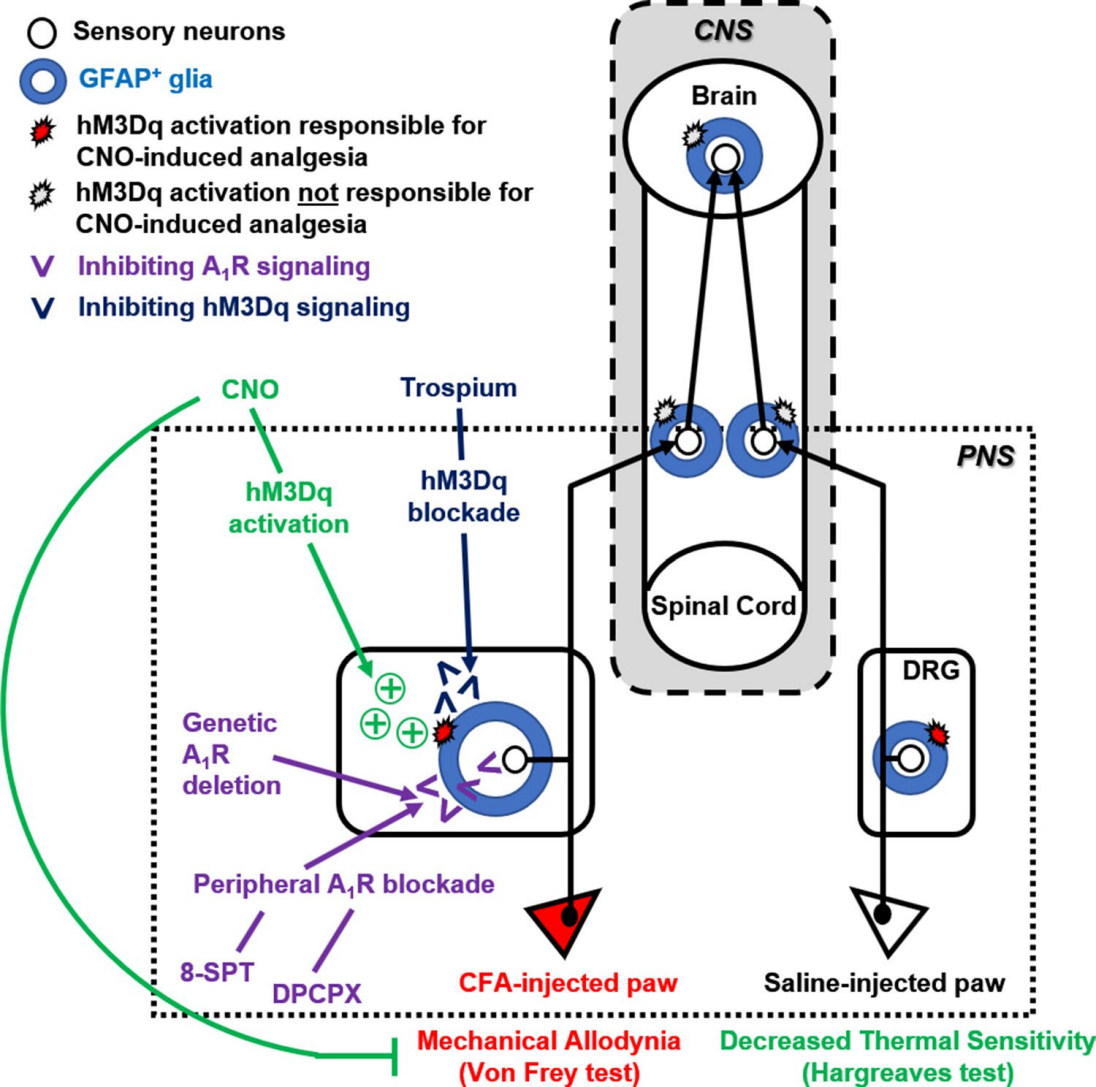
Summary of experimental strategies and approaches. *Gfap*-hM3Dq mice express hM3Dq, a Gq-coupled engineered GPCR in GFAP^+^ glial cells throughout the nervous system. The ligand of hM3Dq, CNO effectively induces hind paw mechanical analgesia (Fig. 1) and decreases thermal sensitivity (Fig. 2) in GFAP-hM3Dq mice. To distinguish the potential analgesic effect of CNS astrocytes vs PNS GFAP^+^ glia following their Gq-GPCR activation, a peripheral acting blocker of muscarinic receptors, Trospium chloride, was administrated to block CNO-induced and hM3Dq-mediated Gq-GPCR activation in peripheral glia. Astrocytic Gq-GPCR activation alone did not lead to significant changes in hind paw mechanical sensitivity (Fig. 3), which suggested that peripheral GFAP^+^ glial Gq-GPCR activation is responsible for CNO-induced hind paw analgesia. The glial-induced analgesic effect was independent from inflammation and not affected by peripheral sympathectomy (Fig. 4), suggesting that peripheral glial Gq-GPCR activation directly modulate sensory neuronal activity and/or afferent excitability. To further test this hypothesis, peripheral acting and selective adenosine receptor A_1_ adenosine receptor antagonists were administrated prior to CNO in GFAP-hM3Dq mice. Blockade of peripheral A1R activation completely abolished peripheral GFAP^+^ glial activation induced mechanical analgesia (Fig. 5); similar results were repeated using A1R KO mice that also express hM3Dq in GFAP^+^ glia (Fig. 6). These experiments strongly suggested that peripheral glial Gq-GPCR activation decreases hind paw mechanical sensitivity via peripheral activation of A1R.

Our findings demonstrate that sensory SGCs are capable of reducing heat sensitivity and reversing the symptoms of inflammation-induced mechanical allodynia following Gq-GPCR activation in vivo, thus, suggesting an analgesic role of these cells (Fig. 7). SGCs are the main type of glia in peripheral ganglia^41,42^. In the dorsal root ganglia (DRGs), SGCs form envelopes around individual neurons^41^, creating distinct functional units consisting of a single sensory neuron and several surrounding SGCs. Sensory SGCs express receptors and transporters of neurotransmitters, ion channels and gap junctions^41^, suggesting their physiological roles in regulating sensory neuronal excitability. Sensory SGCs also express the machinery necessary for releasing neuromodulatory peptides, cytokines, and neurotrophins^41^, indicating their active roles in modulating sensory neuronal activity in both health and disease. However, the ability of sensory SGC signaling to modulate nociception has never been tested in intact and free-moving animals. This is due to the difficulties with selective activation of SGC signaling pathways in vivo. In the current study, we took advantage of the *Gfap*-hM3Dq transgenic mouse line^25^ in which GFAP^+^ glial Gq-GPCR signaling pathways could be selectively activated in vivo by an otherwise bio-inert ligand, CNO^29^. The *Gfap*-hM3Dq mice and Cre-dependent hM3Dq mice have been used in recent studies to assess the physiological roles of peripheral GFAP^+^ glia in vivo^25,27,28,43^. In combination with the pharmacological manipulations, *Gfap*-hM3Dq mice served as a powerful model to study GFAP^+^ glial Gq-GPCR signaling in nociception.

How does SGC Gq-GPCR activation produce analgesic effect on hind paw heat and mechanical sensitivity? Our data suggest that it is likely due to a SGC-mediated inhibitory effect on excitability of DRG neurons via A_1_ adenosine receptor activation. Although our approach did not restrict the genetic deletion or the pharmacological inhibition of A_1_Rs in DRG neurons, purinergic signaling has been reported to mediate SGC-neuron interaction in the DRGs^44^. We established that blocking peripheral adenosine receptors with SPT, or A_1_Rs with CPX, completely reverses CNO-induced analgesia in *Gfap*-hM3Dq mice, suggesting that glial Gq-GPCR activation likely activates neuronal A_1_Rs in the sensory ganglia. These data are in line with the previous results which showed that astrocyte-released adenosine triphosphate (ATP) activates A_1_Rs in hippocampal neurons, leading to heterosynaptic suppression of glutamatergic synaptic responses^45^. We also found that CNO-induced analgesia was completely abolished in A_1_R knockout mice, further confirming that A_1_R activation is responsible for the neuromodulatory effect of SGC Gq-GPCR activation. Interestingly, CNO-induced analgesia remained intact in A_2A_R knockout mice in the context of CFA-induced inflammatory pain. It has been reported that A_2A_R knockout mice exhibit a significant decrease of mechanical allodynia in animal models of neuropathic pain, as well as a suppression of thermal hyperalgesia and allodynia^39^. Taken together, our data suggest that SGC Gq-GPCR signaling modulates sensory neuronal activity exclusively via activation of A_1_R but not of A_2A_R, in inflammation-induced chronic pain models.

The analgesic effect of SGC activation was only suggested once in the previous studies^46^. Chen et al. reported that the P_2_X_7_R activation in sensory SGCs tonically suppressed neuronal P_2_X_3_R expression and decreased the excitability of DRG neurons^46^. P_2_X_7_R is abundantly expressed in sensory SGCs^46–48^, and can trigger exocytosis of ATP via increases in Ca^2+^^46^, suggesting potential inhibitory feedback via bi-directional purinergic signaling between DRG neurons and SGCs. CNO-induced hM3Dq activation also elevates intracellular Ca^2+^ in SGCs. It is possible that hM3Dq activation in sensory SGCs induces ATP release via similar mechanisms as those triggered by P_2_X_7_R activation. However, hM3Dq activation also leads to the activation of many Gq-GPCR signaling cascades in GFAP^+^ glia^49^ in addition to elevating intracellular Ca^2+^. Additional studies are needed to identify the molecular mechanism underlying potential CNO-induced ATP release from sensory SGCs.

Pharmacogenetic activation of central GFAP^+^ glia produces a mild sedative effect^25^ and impaired motor function (Fig. 3E,F), which might influence the perception of pain or the motor function in nociceptive behavior. In this study, trospium chloride, a peripheral hM3Dq blocker, was used to distinguish the effects of central *vs* peripheral glial activation following CNO administration. The trospium chloride blockade of peripheral hM3Dq activation completely abolished CNO-induced analgesia, suggesting that peripheral GFAP^+^ glial activation is responsible for the observed analgesic effect on inflammation-induced mechanical hypersensitivity.

It was important to compare the effect of CNO-induced analgesia to the sympathectomy-produced reversal of mechanical hypersensitivity, as reports indicate that sympathetic sprouting into DRGs contributes to chronic pain following peripheral injury^33,35,50^. However, sympathetic blockade^51^ and chemical sympathectomy^52^ resulted in poor clinical outcomes in patients suffering from chronic pain^53^. Therefore, it was unexpected to observe that 6-OHDA-induced peripheral sympathectomy completely prevented CFA-induced mechanical allodynia in *Gfap*-hM3Dq mice (Fig. 4). CNO administration in *Gfap*-hM3Dq mice led to an additional analgesic effect on mechanical sensitivity in both CFA-injected and saline-injected hind paws (Fig. 4). These data suggest that SGC Gq-GPCR signaling modulates nociception independently of inflammation-induced sympathetic pain mechanisms.

We previously reported that CNO-induced sympathetic ganglionic SGC activation in *Gfap*-hM3Dq mice increased sympathetically-driven cardiac function^27,43^, and blood pressure^25^ in vivo. Our findings suggest that sympathetic SGCs directly increase post-ganglionic neuronal activity following CNO-induced Gq-GPCR activation^27^. However, in the same animal, sensory SGC activation produces a robust analgesic effect that is likely due to direct inhibition of sensory neuronal activity. It is unclear how the same Gq-GPCR activation in sympathetic and sensory SGCs produce opposite neuromodulatory effects. Future studies on the molecular mechanisms underlying ganglionic SGC-neuron interaction in both sympathetic and sensory ganglia are needed.

As reflected in Fig. 7, the current study points towards the SGC Gq-GPCR signaling as a potential target for reversing inflammatory pain. Unpublished data from our laboratory indicate that in sciatic nerve injury-induced neuropathic pain model, CNO i. p. administration also significantly decreases mechanical allodynia in ipsilateral hind paw in *Gfap*-hM3Dq mice. In the current report, we provided data that CNO-induced analgesia occurred in saline-injected and intact hind paws in *Gfap*-hM3Dq mice. Taken together, our findings strongly suggest that SGC Gq-GPCR activation-induced analgesia applies to physiological and pathological conditions, including both nerve-injury-induced and inflammation-induced chronic pain models.

Therapeutic strategies targeting glial activation have been previously considered for pain control^23^. However, the glial inhibitors currently utilized in pain models are usually selected for their anti-inflammatory properties or inhibitory effects on glial metabolism. The choice of glial inhibitors is mostly based on the assumption that glial activation is pro-nociceptive^23^. Our data in *Gfap*-hM3Dq mice demonstrate a strong anti-nociceptive property of Gq-GPCR signaling pathways in sensory SGCs. For several reasons, we believe that sensory SGC Gq-GPCR signaling pathway could be a strong candidate for potential pharmacological therapies for the treatment of acute and chronic pain. First, glial-mediated inhibition of sensory neurons can reduce the ongoing peripheral nociceptive input into the CNS, which is required for the maintenance of chronic pain^54,55^. Second, sensory SGCs are positioned near the spinal cord, therefore, pharmacological activation of SGC signaling would not require drugs to cross the blood–brain barrier. Third, modulation of sensory neuronal activity via glial signaling may reduce the potential side effects of direct activation of the receptors on neuronal cell bodies. Finally, the pharmacological effects of glial Gq-GPCR activation in vivo are fast-acting and long-lasting^25,27^, despite the rapid clearance of the ligand^56^. The combination of the above-mentioned factors makes SGC an ideal candidate for developing glia-focused therapeutic approaches. Future efforts aimed at screening for glial-specific Gq-GPCRs or chemical compounds to activate glial Gq-GPCRs would provide additional resources for the development of novel and effective treatments for chronic pain conditions.

## Materials and methods

### Animals

All transgenic and knockout mice were generated and maintained in the C57BL/6J background. Males were used in all experiments. Mice were maintained in a temperature-controlled environment at the University of North Carolina at Chapel Hill (UNC-CH) on a 12-h light/12-h dark cycle, with ad libitum access to food and water. Experiments took place during the light cycle. All experimental procedures on animals were performed according to the protocols approved by the IACUC of the UNC-CH (protocol 13-276.0).

*Gfap*-hM3Dq mice were generated, bred, and genotyped as previously described^25^. Briefly, the mice expressed HA-hM3Dq driven by a 2.2kB human GFAP promoter. *Gfap*-hM3Dq^+/−^ mice were bred to C57Bl6/J wild type (WT) mice (Jackson Labs, Bar Harbor, ME) to generate hemizygous and WT progeny. All experiments were performed in *Gfap*-hM3Dq^+/−^ mice (hM3Dq mice) from N11 to N15 generational backcross. In all experiments, hM3Dq-negative, WT littermates were used as controls.

Mice lacking A_1_R (A_1_^−/−^ mice)^57^ and A_2A_R (A_2A_^−/−^ mice)^58^ were gifted by Dr. Stephen Tilley (UNC-CH). These mouse lines were crossed separately with *Gfap*-hM3Dq^+/−^ mice, resulting in hemizygous hM3Dq expression in GFAP^+^ cells in either A_1_^−/−^ mice (*Gfap-*hM3Dq^+/−^::A_1_^−/−^) or A_2A_^−/−^ mice (*Gfap-*hM3Dq^+/−^::A_2A_^−/−^). In all presented experiments, *Gfap*-hM3Dq^+/−^ mice with heterozygous expression of A_1_R (*Gfap*-hM3Dq^+/−^::A_1_^+/−^ mice) or A_2A_R (*Gfap*-hM3Dq^+/−^::A_2A_^+/−^ mice) were used as controls, respectively.

### Complete Freund’s adjuvant (CFA) induced inflammatory pain

To induce acute inflammation and subsequent chronic pain, 20 µL CFA (Sigma-Aldrich, MO, USA) was injected into the right hind paw of each mouse. Saline (20 µL) was injected into the left hind paw as an internal experimental control. CFA-injected mice were placed on soft bedding for 7 days during the rest of the experimental period. The mechanical sensitivity of the CFA-injected and saline-injected paws was assessed before CFA injections and 5 or 7 days after CFA injections depending on the experiments (Figs. 1A, 3A, 4A).

### Von Frey test for assessing mechanical pain threshold

The Von Frey test was used to determine the mechanical pain threshold of the hind paws of hM3Dq mice and their WT littermate controls, as previously described^59^. The apparatus consisted of a clear plastic isolation chamber (7.5 cm wide × 7.5 cm long × 15 cm tall) on top of a wire mesh floor (0.5 cm^2^ grid) to allow access to the hind paws. Mechanical threshold was measured as the minimum filament weight that induced 5 paw responses (withdrawal or licking/biting) out of 10 applications. For each mouse, a baseline threshold was established just prior to CFA injection. Five days after CFA injection, pharmacological intervention of pain threshold was tested before and after pharmacogenetic (CNO administration) or pharmacological manipulation in 15-min increments.

### Hargreaves test for assessing thermal pain threshold

*Gfap*-hM3Dq mice and their WT littermate controls were individually placed in a plastic isolation container (7.5 cm wide × 7.5 cm long × 15 cm tall) on an elevated glass plate. After 60 min of habituation, a low intensity thermal beam was applied to the footpad of each hind paw intermittently in time increments, as indicated in Fig. 2. The latency time from the application of the light beam to the paw flick response was recorded. The beam was cut off after a maximum of 20 s to prevent tissue damage. CNO was administered by either intraperitoneal injection (i. p., 0.25 mg/kg) or intrathecal injection (i. t., 0.50 mg/kg) using the direct lumbar puncture method, as previously described^60^.

### Rotarod test for assessing motor function and learning

The balance and motor coordination of *Gfap*-hM3Dq mice and their WT littermate controls were tested on an accelerating rotarod, as previously described^25^. Speed (RPM) was set at an initial value of 3, with a progressive increase to a maximum of 30 RPM during a 5-min trial. CNO (0.25 mg/kg) was given 15 min prior to the first trial. Mice were pretreated with saline or 20 mg/kg trospium^27^ 15 min prior to injection of CNO. Each mouse underwent a training session consisting of three trials, with 45 s between each trial. The latency time to fall, or to rotate fully around the turning barrel, was recorded.

### Chemical sympathectomy

Chemical sympathectomy was performed by two 6-hyadroxydopamine (6-OHDA; 150 mg/kg in 0.1% ascorbic acid, i. p.) injections three days apart^36^ to trigger sympathetic denervation in peripheral organs including sensory ganglia. The α-adrenergic receptor blocker phentolamine (150 µg/kg, i. p.) was injected with the first 6-OHDA injection to protect animals from massive release of catecholamines associated with 6-OHDA administration^36^. Saline-injected *Gfap*-hM3Dq animals received two 0.1% ascorbic acid injections, and a phentolamine injection with the first ascorbic acid injection. This treatment regimen has been proven effective in eliminating sympathetic nerve-released catecholamines^27,36,38^. Von Frey tests were performed 3 days after the second 6-OHDA injection^27^.

### Pharmacogenetic and pharmacological manipulations

Clozapine-*N*-Oxide (CNO, powder form, provided by the NIH through B.L. Roth) was dissolved in DMSO at 1 mg/mL, and then diluted in physiological saline to applicable concentration. CNO was administered i. p. at 0.25 mg/kg^25,27^ (0.125% final DMSO concentration), unless otherwise indicated. Trospium Chloride (Toronto Research Chemicals) was diluted in physiological saline and administered i. p. at 20 mg/kg^27^. 8-(*p*-Sulfophenyl) theophylline hydrate (SPT) and 8-Cyclopentyl-1,3-dipropylxanthin (CPX) were purchased from Sigma Aldrich (Milwaukee, WI). Each drug was initially dissolved in DMSO (10 mg/mL), and then diluted to working concentration with physiological saline. The non-selective, blood–brain-barrier impermeable adenosine receptor antagonist SPT was delivered at 10 mg/kg^61^. The selective A_1_R antagonist CPX was delivered at 1 mg/kg^61^.

### Statistics

The basal sensitivity between genotypes in the Hargreave’s test was analyzed using two-tailed independent samples t-test. All other experiments were analyzed using two-way ANOVA with genotype or drug as between subjects factors, and time as a repeated measure, with Fisher’s Least Significant Difference (LSD) post-hoc analysis, as needed. Statistics were performed using Statistica 12 (Dell, Tulsa, OK), and the figures were generated using Graphpad Prism 7.04 (La Jolla, CA; https://www.graphpad.com/scientific-software/prism/).

### Statement of ethical approval

The study was approved by the Institutional Animal Care and Use Committee (IACUC) of the UNC-CH (protocol 13-276.0). All experiments were performed exclusively in UNC-CH and in accordance with the relevant guidelines and regulations.
